# Analyzing Facial and Eye Movements to Screen for Alzheimer’s Disease

**DOI:** 10.3390/s20185349

**Published:** 2020-09-18

**Authors:** Uiseo Nam, Kunyoung Lee, Hyunwoong Ko, Jun-Young Lee, Eui Chul Lee

**Affiliations:** 1 Department of Computer Science, Graduate School, Sangmyung University, Seoul 03016, Korea; uiseo0225@gmail.com (U.N.); guy9284@gmail.com (K.L.); 2 Interdisciplinary Program in Cognitive Science, Seoul National University, Seoul 08826, Korea; powerzines@snu.ac.kr; 3 Department of Psychiatry, Seoul National University College of Medicine & SMG-SNU Boramae Medical Center, Seoul 03080, Korea; 4 Department of Human-Centered Artificial Intelligence, Sangmyung University, Seoul 03016, Korea

**Keywords:** dementia, eye movement, face, facial movement, Alzheimer’s disease, gaze correlation

## Abstract

Brain disease can be screened using eye movements. Degenerative brain disorders change eye movement because they affect not only memory and cognition but also the cranial nervous system involved in eye movement. We compared the facial and eye movement patterns of patients with mild Alzheimer’s disease and cognitively normal people to analyze the neurological signs of dementia. After detecting the facial landmarks, the coordinate values for the movements were extracted. We used Spearman’s correlation coefficient to examine associations between horizontal and vertical facial and eye movements. We analyzed the correlation between facial and eye movements without using special eye-tracking equipment or complex conditions in order to measure the behavioral aspect of the natural human gaze. As a result, we found differences between patients with Alzheimer’s disease and cognitively normal people. Patients suffering from Alzheimer’s disease tended to move their face and eyes simultaneously in the vertical direction, whereas the cognitively normal people did not, as confirmed by a Mann–Whitney–Wilcoxon test. Our findings suggest that objective and accurate measurement of facial and eye movements can be used to screen such patients quickly. The use of camera-based testing for the early detection of patients showing signs of neurodegeneration can have a significant impact on the public care of dementia.

## 1. Introduction

In the 21st century, mortality and fertility rates have been declining, while medical and living standards have been increasing. As a result, we are facing a population aging problem [1]. Forms of dementia, such as Alzheimer’s disease, are mostly caused by aging and degenerative brain changes, and, unfortunately, incidence and social costs have rapidly increased since 2010 [2]. Due to the progression of the disease, the brain’s functioning deteriorates, making it difficult to maintain an individual’s life independently, as most patients experience self-loss and are unable to participate in everyday experiences [3]. Early detection is very important for dementia patients, as they are dependent; it can reduce the caregiver’s burden and social costs. Therefore, there is a pressing need for a method that can easily and quickly detect dementia.

Recently, increasingly more studies have shown that recognition of emotions or measurement of mental states can be achieved by analyzing eye movements [4,5,6]. Although subtle changes in eye-movement patterns are difficult to identify, they can reveal the emotion or mental state that the brain is processing because their structural characteristics are linked to the brain. Dementia is commonly known as a degenerative brain disease that causes brain damage, cognitive impairment, and behavioral and psychological symptoms [7,8]. In addition, many patients experience exhibit visual neurological symptoms and signs [9]. However, it is very difficult to evaluate the visual acuity of dementia patients; doing so depends on the pathological proficiency of the diagnosing doctor [10]. Therefore, in this study, we analyzed the facial and eye movement patterns of patients with Alzheimer’s disease and cognitively normal people when they were watching videos to identify the symptoms of dementia.

Previous research on the subject can be categorized into two groups: studies that used eye-feature-based methods and studies that used facial-feature-based methods. One method for measuring cognitive responses and brain function that uses the human eye is the pupillary response. Another method is gaze analysis [11,12,13]. When degenerative brain change makes it impossible for a stimulus that enters the retina to be processed, the pupil cannot respond to that stimulus [14]. As such, a method that uses pupil dilation and fixation duration has been proposed for analyzing the human mental state and the recognition of emotion [15]. 

Several studies have examined pupillary response. One study analyzed the symptoms of post-traumatic stress disorder (PTSD) by measuring changes in the pupil size and fixation duration of Iraqi veterans when viewing traumatic and neutral images. When viewing trauma-related stimuli, higher PTSD levels were associated with vigilance rather than avoidance, but this was not found in those with lower PTSD levels [16]. Another study suggested that early markers of Alzheimer’s disease could be identified in the pupil responses by analyzing the correlation between the pupil dilation when participants perform cognitive tasks and the cognitive decline. Granholm and colleagues found that the pupil expanded according to the needs of the task in patients with mild cognitive impairment (MCI) and cognitive impairment when performing a task that required cognitive function. However, they also found that pupil size and performance tended to decrease dramatically when tasks were demanding and exceeded the patient’s cognitive abilities [17]. 

Other studies measured only the gaze and did not examine the pupil in psychiatric disorders. Weeks et al. performed some experiments on patients with social anxiety disorder. The results confirmed that a gaze characteristic or response for people with anxiety is avoidance. They tracked the participants’ eyes when they were watching videos that could trigger social anxiety. The gaze avoidance of participants with social anxiety disorder was proportional to their anxiety and fear of the stimuli [18]. In another study, MCI or dementia patients watched videos according to some given conditions, and it proved possible to infer cognitive functions by recording and analyzing the participants’ eye movements and observing the changes in their gaze patterns [19]. One research study developed a model that can detect the symptoms of depression using various features, such as head pose, facial expression, eye gaze, and audio features. In this model, a feature point is specified by extracting a landmark from a region of a face, and the facial expression and pose features are extracted based on the distance between the feature points and the velocity of the movement. The extracted features are classified using a support vector machine and neural networks [20].

However, there are problems in the existing studies due to the complexity of the experiment, the restricted environment, and the gaze analysis method. For example, participants completed subjective assessments before and after the experiment and performed a task that met the conditions within a restricted environment. This approach increases the complexity of the experiment. Dementia patients have a decline in attention and concentration. Moreover, they often experience vigilance or anxiety. Negative emotions can also affect the patient’s cognitive function. Therefore, experiments that involve complex tasks can intensify the symptoms, potentially affecting the results. This makes it difficult to make accurate diagnoses and to implement models that can be used with actual patients.

Another problem with the gaze analysis method is that of determining how to define the gaze. We consider that the gaze cannot be defined only by the direction or velocity of the eye movements, because the gaze reflects human emotions or mental states; that is, the behavioral aspects of the natural gaze should include not only the eyes but also the facial movements. Thus, in this study, we analyzed the correlation between the facial and eye movements of patients with Alzheimer’s disease while they were watching videos. Based on these results, a method is provided that enables swift and accurate analysis of eye movements of patients with Alzheimer’s disease.

## 2. Materials and Methods

### 2.1. Participants

The experiment was conducted with elderly people over 60 years old who were divided into two groups. One group, the mild Alzheimer’s disease group, consisted of 17 patients with mild Alzheimer’s. The diagnosis of probable Alzheimer’s disease was made according to the criteria of the National Institute of Neurological and Communicative Disorders and Alzheimer’s Disease and Related Disorders Association (NINCDS-ADRDA) [21]. The exclusion criteria for participation were as follows: any depressive symptoms; impaired physical mobility that might influence the study process; history of neurological disease (i.e., head trauma or stroke) or major psychiatric disease according to the criteria of the Diagnostic and Statistical Manual of Mental Disorders, Fourth Edition (DSM-IV) [REF]; auditory or visual difficulties that could disrupt the experiment procedure; refusal to give consent; and inability to properly complete the test as judged by an examiner. Exclusion criteria were determined by a board-certified psychiatrist (J.Y.L.). The severity of dementia was rated by Clinical Dementia Rating scale, and the Clinical Dementia Rating scores of the patient group were 0.5 (very mild) or 1 (mild) [22]. The other group, which was the normal cognitive group, included participants without dementia or other mental disorders. This group consisted of 17 healthy participants. Information about the two groups (Alzheimer’s disease and normal cognition) who participated in this research can be found in Table 1. In the table, education refers to the number of years that the participant had been educated. The Mini-Mental State Examination (MMSE) is based on a perfect score of 30. The comparison of differences between the two groups for the MMSE score is significant: normal cognitive > Alzheimer’s disease (*p* < 0.001, regardless of age or sex).

### 2.2. Ethical Considerations

All participants gave their informed consent before taking part in the study. The study was conducted in accordance with the Declaration of Helsinki. It was approved by the Institutional Review Board of SMG-SNU Boramae Medical Center (IRB No.30-2017-63).

### 2.3. Experimental Configuration

**Figure 1:**
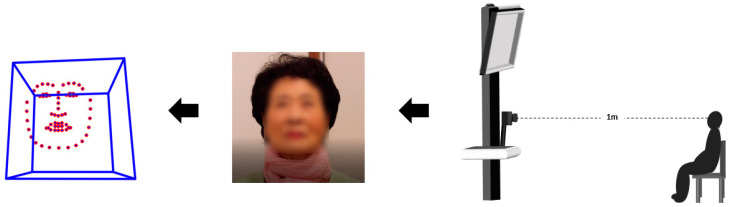
Example of the experimental environment.

The video used in this experiment was about 22 min long and consisted of six different short films that evoked the six basic emotions (happiness, sadness, fear, anger, surprise, and disgust). Participants watched each short film for about 1 min, and the participants then rested for 2 min. After 2 min of rest, they watched the next video. There was no difference in individual responses to the six short films. The face video data used in this study were obtained by recording the face of the participant while they watched the video. A Canon EOS 70D camera (Canon Inc., Tokyo, Japan) was used to record at 30 frames per second with a 1920 × 1080 resolution. As shown in Figure 1, the distance between the camera and the subject was set to 1 m. We conducted the experiment individually in a separate room. In addition, for the consistency of the experiment’s environment, we isolated the participants from factors, such as light, motion, and background, that could have obstructed the participant’s concentration.

### 2.4. Image Analysis

**Figure 2:**
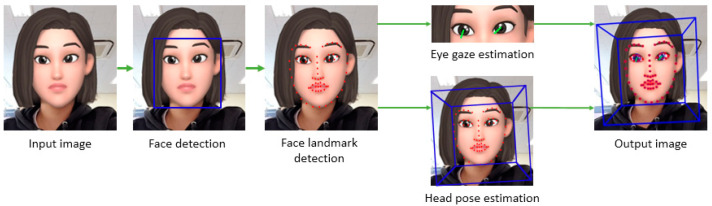
Facial and eye movement extraction by OpenFace 2.0 [23].

**Figure 3:**
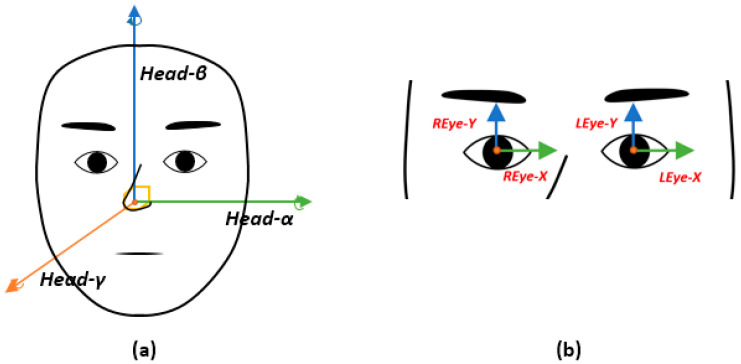
Coordinate axes: (**a**) face and (**b**) eye.

To acquire the eye and facial movement data of an Alzheimer’s patient, the facial and eye coordinate values were first extracted from the participant’s video. For the extraction, we referred to the method shown in Figure 2, which was taken from a facial behavior analysis toolkit called OpenFace 2.0 [23]. The OpenFace 2.0 toolkit provides an open source library for detecting faces, extracting facial landmarks based on facial features, and estimating eye gaze and head pose. It also includes the process of recognizing the action units of the face. In this study, only the process of estimating the head pose and eye gaze was used [24,25,26,27]. The extracted values are output together in a comma-separated values (.csv) file format along with the resulting processed image. The gaze value is a vector value that reflects the amount of change in the feature point movement of the face landmark [25,26,27]. Figure 3 presents the coordinate axes used in this study. The facial coordinate axes used were Head-α, Head-β, and Head-γ, which represented the rotation of the facial movements on the 3D axis, as shown in Figure 3a. As for the eyes, the left eye and right eye coordinate axes were REye-X, REye-Y, LEye-X, and LEye-Y, which were the moving coordinate values on the 2D axis, as shown in Figure 3b.

### 2.5. Statistical Analysis

#### 2.5.1. Spearman’s Correlation Coefficients of Facial and Eye Movements

**Figure 4:**
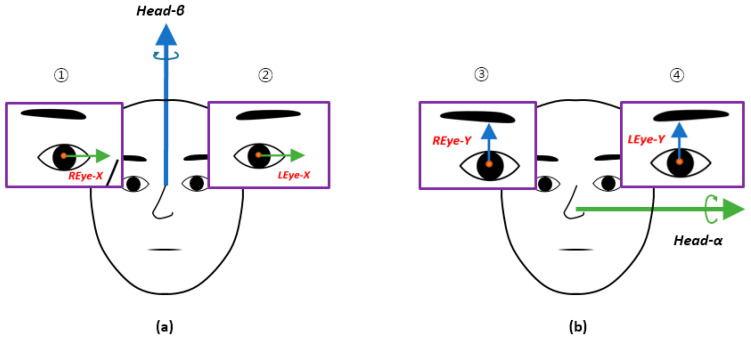
A pair of axes was used to obtain the correlation coefficient: (**a**) horizontal and (**b**) vertical.

Using the extracted coordinate values, Spearman’s correlation coefficients were calculated to analyze the correlations between the vertical and horizontal facial movements and eye movements [28,29]. The coordinate axis pair was used to identify the correlation between the two directions, as shown in Figure 4a (a pair of axes was used to obtain the correlation in the horizontal direction). The eyes in the horizontal direction used the X-axis. Since the face uses a rotation axis, the vector values in the horizontal direction were obtained using the Head-β axis (Figure 4b shows the axis used to find the correlation between the facial and eye movements in the vertical direction). For the vertical direction of the face, we obtained the vector value by using the rotation Head-α axis. Thus, four types of correlation coefficients were obtained for each group using combinations of 1 to 4, as presented in Figure 4.

#### 2.5.2. Mann–Whitney–Wilcoxon Test of the Spearman’s Correlation Coefficient

We used the Mann–Whitney–Wilcoxon (MWW) test to determine if the two groups had different or equal correlations [30]. Before performing the MWW test, we conducted the Kolmogorov–Smirnov (KS) test to examine the normality of the four types of correlation coefficients obtained from the two groups [31].

The KS test showed that the distribution of the correlation coefficient was non-normal in both the Alzheimer’s disease and normal groups. Based on this result, the MWW test was performed to test the difference between the two independent groups. All tests were performed using the significance level of 95% (*p* > 0.05).

## 3. Results

**Figure 5:**
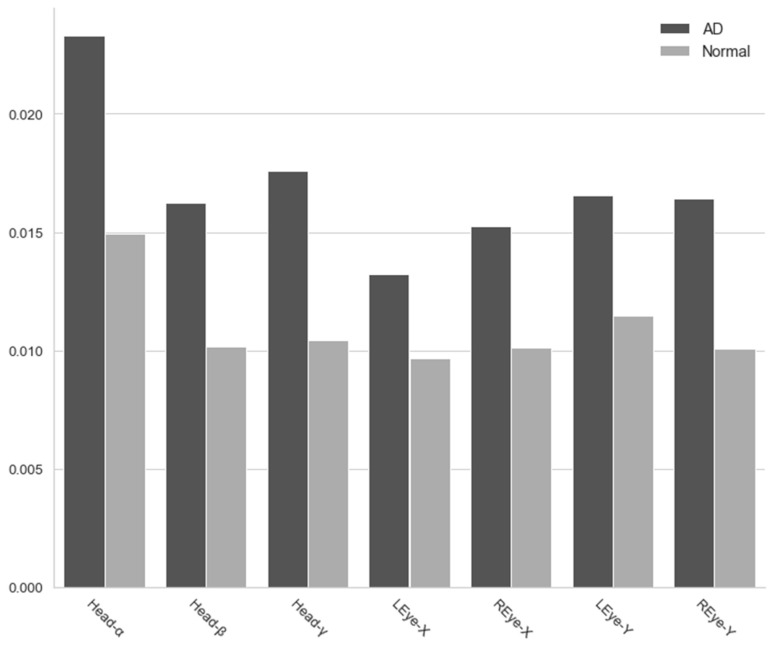
Variance for movement in all directions.

We found that the gaze variance in the Alzheimer’s disease group was significantly greater in all directions than in the normal group. One of the symptoms that comes with cognitive decline is a decrease in concentration, which causes frequent eye movements and facial movements. The degree of dispersion can be seen in Figure 5.

**Figure 6:**
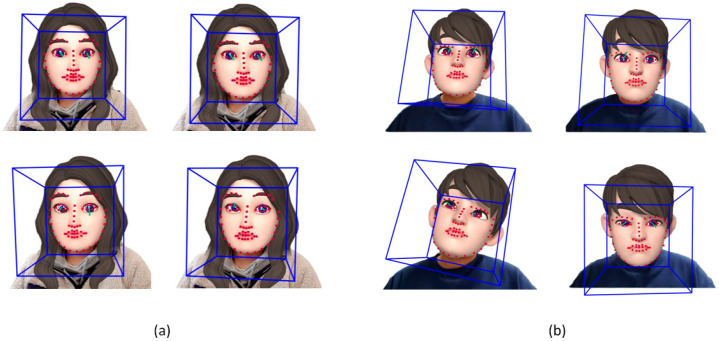
Extracted facial landmarks: (**a**) normal group and (**b**) AD group.

Figure 6 displays some of the extracted facial landmarks. The Alzheimer’s patients made many facial movements, and the directions of their face and eyes coincided in the vertical direction. The normal group had few facial movements, and we did not find any pattern of facial and eye movements in the vertical direction.

**Figure 7:**
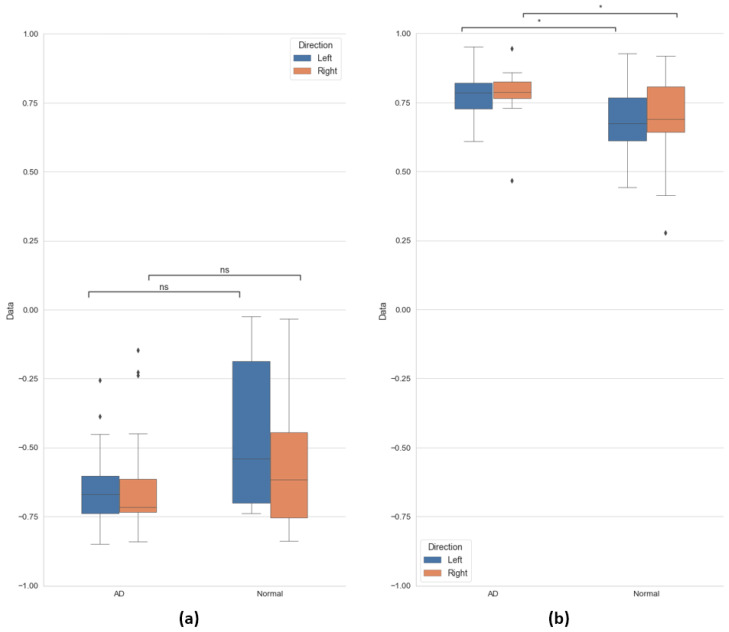
Correlation coefficients represented by the boxplots: (**a**) horizontal and (**b**) vertical.

The horizontal correlation coefficient was not significantly different between the two groups, and it was negatively correlated. That is, when the face moved horizontally, the eyes tended to move away from the face. The distribution of these correlation coefficients is shown in Figure 7a.

As shown in Figure 7b, the correlation coefficients for the vertical direction were different between the two groups. In the Alzheimer’s disease patient group, the correlation coefficient in the vertical direction is composed of values close to 1, indicating a positive correlation. In the case of the normal group, the correlation coefficient values in the vertical direction have a wider distribution than the Alzheimer’s disease patient group, and there is no consistency of gaze within the group. In conclusion, in the case of the Alzheimer’s patients, when the face moved up and down, the eye movement tended to move in the same direction as the face, whereas in the normal group, the facial movement did not correlate with the direction of the eyes.

Table 2 shows the statistics of the correlation coefficients obtained. Using data from all participants, “direction” refers to the eyes in pairs of facial and eye axes (see Figure 4).

The MWW test results verifying the significance of the two groups are also shown in Figure 7. For the *p*-values and annotation of the MWW test shown in the figure, refer to Table 2.

## 4. Discussion and Conclusions

### 4.1. Variance for Movement in All Directions

As a result of comparing the degree of variation in the movements of the Alzheimer’s patient and the healthy person in each direction, we found that the degree of variation was higher in the Alzheimer’s disease patient group. That is, the face and eyes moved more often in multiple directions in patients with Alzheimer’s disease. One type of abnormal eye movement caused by dementia is distraction [32]. Patients with Alzheimer’s disease have difficulty looking upwards and have poor visual fixation. Moreover, during a visual search, the target object often cannot be found, and the gaze duration for other objects is long because it lacks a specific focus [33].

When cognitive ability deteriorates, the ability to concentrate attention decreases once the target object is detected, and the amount of eye movement increases. Researchers argue that these variations in gaze in Alzheimer’s patients are due to damage to frontal and parietal lobes; deficits in these areas are known to be related to dysfunctional attention in the course of Alzheimer’s disease, leading to deficits in the initiation and suppression of saccades and smooth pursuit [34]. Patients with Alzheimer’s disease may have a higher degree of facial movement in order to overcome these problems. Accordingly, the above-mentioned symptoms in the existing eye-movement screening method displayed in the group of patients with Alzheimer’s disease may be the clinical manifestation owing to the reduced attention. In this respect, our results suggest that the abnormal eye movements measured in the current study can be a marker in screening for Alzheimer’s disease.

### 4.2. Correlation in Vertical Direction between Face and Eye Movements

Dementia is commonly referred to as a symptom associated with the cognitive impairment of a neurodegenerative disease. Abnormal eye movements appear in most types of dementias [35]. We found that the vertical gaze of Alzheimer’s patients differed from the gaze of the normal group, using only the camera’s records. These patients had a statistically significantly higher correlation coefficient between eyes and face in the vertical direction, despite a higher degree of change in face and eye movement.

Vertical eye movement paralysis refers to abnormal saccadic eye movement in the vertical direction, which is related to the results of this study. Deficits in the movement of visually guided vertical saccades in Alzheimer’s disease may be related to the attentional impairment, rather than to the ocular motor nerve impairment. Our study’s results indicate that the face and eyes may move relatively simultaneously due to reduced vertical smooth eye movement [36].

Dementia is difficult to diagnose accurately because the boundary of symptoms is difficult to quantify. To check whether or not the existing eye movement is abnormal, the patient is asked to move the eye according to the given condition, and a score is given when executed properly. During the test, the patient uses only eye movements, which allows the test-giver to ensure that eye movements exceed the normal range compared to the cognitively normal group [37]. However, even in the cognitively normal group, there are cases where results are out of range due to aging; the movements of the eye are very discreet and are displayed only when the range is greatly exceeded. Hence, early diagnosis is difficult [38].

### 4.3. Limitations and Future Directions

Given the normality test process and the amount of data, appropriate statistical methods were applied to obtain results sufficient to demonstrate the difference between the two groups participating in the experiment. However, more data are needed for such analysis to be applied to existing dementia diagnostic methods. In addition, differing types of features need to be analyzed to diagnose the exact disease among neurodegenerative diseases that indicate dementia.

The analyses used in the current study were correlation and variance along each direction of facial and eye movement. It is important to identify dementia accurately; although patients’ general symptoms may be similar, the degree of damage and its progress in certain areas of the brain depends on the disease [38]. To overcome these limitations, future studies will explore ways in which additional data from groups with Alzheimer’s disease and cognitively normal groups can be collected. Furthermore, we are looking for a method that can classify different features of eye movements and facial movements depending on the disease. We are investigating the method of using feature points in the landmark extraction method used in the current research. We are also considering ways to classify various fine features using machine learning.

### 4.4. Conclusions

Alzheimer’s disease has severe cognitive symptoms. In patients with dementia, early detection can have a significant impact on their care. Therefore, it is very important to detect and treat the symptoms of dementia immediately and efficiently. Our study analyzed the differences in gaze between patients with Alzheimer’s disease and cognitively normal people in an intuitive way, and the findings suggest these differences can be used to screen Alzheimer’s disease symptoms quickly using only camera recordings. In addition, we propose a method that enables objective and accurate measurement of eye movements by determining the correlation between facial and eye movements. The ultimate goal of our study is to provide a foundation for the development of automated tools for analyzing the symptoms of dementia patients. Further symptom analysis and research should increase the accuracy and significance of the symptom diagnosis and build an automated system based on the measured data by incorporating deep learning.

## Figures and Tables

**Table 1:** Characteristics of the research participants.

Variable	AD (*n* = 17)			Normal (*n* = 17)		
	Mean	*SD*	%	Mean	*SD*	%
Age	77.23	6.79	-	74	6.53	-
Education ^1^	9.7	3.94	-	10.94	5.06	-
Gender ^2^	-	-	47/53	-	-	41/59
MMSE	20.12	5.28		27	2.85	

^1^ Years of education; ^2^ male/female. AD = Alzheimer’s disease. Normal = cognitively normal. *SD* = standard deviation.

**Table 2:** Results of Spearman’s correlation.

	Horizontal (a)				Vertical (b)			
**Direction**	Left		Right		Left		Right	
Group	AD	Normal	AD	Normal	AD	Normal	AD	Normal
Median	−0.669	−0.54	−0.716	−0.616	0.785	0.673	0.788	0.69
IQR	0.135	0.515	0.118	0.309	0.092	0.156	0.06	0.163
Mean ± *SD*	−0.639 ± 0.162	−0.467 ± 0.219	−0.62 ± 0.25	−0.557 ± 0.223	0.787 ± 0.08	0.681 ± 0.132	0.783 ± 0.157	0.68 ± 0.096
MWW	*U*	Sig	*U*	Sig	*U*	Sig	*U*	Sig
	91	0.067	120	0.408	213	0.019 (*)	210	0.025 (*)

* 0.01 < *p* ≤ 0.05. AD = Alzheimer’s disease. Normal = cognitively normal. SD = standard deviation.
